# Radiation therapy of anal canal cancer: from conformal therapy to volumetric modulated arc therapy

**DOI:** 10.1186/1471-2407-14-833

**Published:** 2014-11-18

**Authors:** Angelo Tozzi, Luca Cozzi, Cristina Iftode, Annamaria Ascolese, Maria Concetta Campisi, Elena Clerici, Tiziana Comito, Fiorenza De Rose, Antonella Fogliata, Ciro Franzese, Pietro Mancosu, Piera Navarria, Stefano Tomatis, Elisa Villa, Marta Scorsetti

**Affiliations:** Department of Radiotherapy and Radiosurgery, Istituto Clinico Humanitas Cancer Center, Rozzano, Milan, Italy; Medical Physics Unit, Oncology Institute of Southern Switzerland, IOSI, Bellinzona, Switzerland

**Keywords:** Anal canal, VMAT, RapidArc, Radiotherapy, 3DCRT

## Abstract

**Background:**

To appraise the role of volumetric modulated arc (RapidArc, RA) in the treatment of anal canal carcinoma (ACC).

**Methods:**

A retrospective analysis has been conducted on 36 patients treated with RA since 2009 comparing outcome against a group of 28 patients treated with conformal therapy (CRT). RA treatments were prescribed with SIB technique with 59.4 Gy to the primary tumor and nodes and 49.5 Gy to the elective nodes. CRT was sequentially delivered with 45 Gy to the pelvic target and a boost of 14.4 Gy to the primary tumor.

**Results:**

Median age of patients was 65 yrs for RA (59 yrs for CRT); 90% had Stage II-III (93% in the CRT group). No statistically significant differences were observed concerning survival or control. 5 yrs disease specific survival was 85.7% and 81.2%, loco-regional control was of 78.1% and 82.1% for RA and CRT respectively. RA treatments lead to lower incidence of higher grade of toxicity events (all retrospectively retrieved from charts as worse events). Grade 2–3 toxicity, compared to CRT, reduced from 89% to 68% for GI, from 39% to 33% for GU and from 82% to 75% for the skin. Late toxicity was as follows: 5/36 (14%) and 3/36 (8%) patients had G1 or G2 GI toxicity in the RA group (1/28 (4%) and 4/28 (14%) in the CRT group). GU late toxicity was observed only in 4/28 (14%) patients of the CRT group: 3/28 (11%) had G2 and 1/28 (4%) had G1.

**Conclusions:**

RA treatments of ACC patients proved to be equally effective than CRT but it was associated to a reduction of toxicity.

## Background

The treatment of squamous cell carcinoma (SCC) of the anus evolved in the last decades from the concept of abdomino-perineal resection to the approach of definitive pelvic radiotherapy combined with chemotherapy. The latter is the current standard of care and provided excellent results in term of sphincter preservation, loco-regional control and overall survival. However, this treatment regimen, is often associated with relevant acute dermatological, genitourinary and gastrointestinal toxicities.

From a technical point of view, the use of intensity modulated radiation therapy (IMRT), appeared attractive due to its potential to reduce acute and chronic treatment-related toxicity and it was recently implemented also for the treatment of the anal cancer. IMRT is the delivery of non-uniform photon beams from different entry portals to generate an highly uniform target irradiation with the maximization of the sparing of the surrounding healthy tissues, This method of irradiation resulted in lower rates of acute and late grade > 3 toxicity while maintaining at least similar outcomes in terms of local control and survival as reported in several studies [1–10].

Volumetric modulated arc therapy (VMAT), is a method to combine rotational therapy techniques with intensity modulation and has been investigated extensively and applied to a large variety of clinical indications. RapidArc (RA) is a specific form of VMAT implemented at planning and delivery level with continuous modulation of multileaf collimator, dose rate and gantry rotational speed dynamics. Little has been done so far in testing its role in the radiation treatment of anal SCC. Two planning studies [11, 12], reported about a comparison between IMRT and RA and demonstrated the technical feasibility of RA in terms of improved organs at risk sparing but did not addressed any comparison against conventional conformal therapy, the treatment of choice in many institutes for this category of patients.

Aim of the present report is to summarize the retrospective clinical experience of a single institute over two cohorts of patients treated either with conformal radio-therapy (CRT) or with volumetric modulated arc therapy. CRT, i.e. the delivery of uniform photon beams from multiple entry portals with good homogeneity of target irradiation but limited potential for healthy tissue sparing, was the standard treatment of choice until 2008 for all these patients while, after its clinical introduction, RA became the consolidated technique.

## Methods

Between January 2006 and May 2013, 64 patients with histologically confirmed anal SCC and good performance status (PS 0–1) underwent radiation therapy alone or concurrent chemotherapy. All patients were treated in compliance with the Helsinki Declaration. This study is a summary of a retrospective analysis to the treatment charts and did not required ethical approval pending local regulations.

All patients underwent digital rectal examination, either rigid proctoscopy or flexible sigmoidoscopy, and computed tomography (CT) scans of the abdomen and pelvis for staging. Magnetic resonance imaging and/or endoscopic ultra sound and FDG PET-CT scan were not routinely performed for staging. Patients were staged according to the American Joint Committee on Cancer (AJCC) 2006 guidelines.

Human immunodeficiency virus (HIV) and Human papilloma virus (HPV) viral status and other co-morbidities were recorded to complement the staging information.

Two Clinical Target Volumes (CTVs) were defined on the planning CT images: CTV_boost included the gross tumour volume (GTV) plus a margin of 10 mm to include areas at risk of microscopic spread. These latter were represented by the entire anal canal, the peri-anal region and the meso-rectum. If present, positive lymph nodes were included in the CTV-boost.

The pelvic CTV was contoured by an expansion of 10 mm around the inguinal, femoral, external iliac, internal iliac and common iliac vessels. Muscles and bones were subtracted from the expansion. Contouring was performed in accordance with institutional and international guidelines [13–15]. In some patients, the use of PET-CT imaging improved the identification of the targets for radical dose prescription in the cases eligible to prophylactic irradiation. Planning target volume (PTVs) were contoured by adding an isotropic expansion of 10 mm to the CTVs.

The small bowel, the bladder and the femoral heads as well as the external genitals were contoured as organs at risk. Clinical planning objectives (used as a guidance in CRT and as optimization objectives for RA) were: V_40 Gy_ < 50% for the bladder, V_40 Gy_ < 30% for the bowel (defined as the entire bowel “bag”), V_40%_ < 25% for the genitals; V_40 Gy_ < 25% and D_1%_ < 50 Gy for the femoral heads (V_x Gy_ is the volume of a structure receiving at least x Gy while D_x%_ is the dose received by at maximum x% of an organ).

All treatment plans were developed using the Varian Eclipse planning system and dose calculation was performed with the Anisotropic Analytical Algorithm with a spatial resolution of 2.5 mm. RA plans were optimized with the Progressive Resolution Algorithm (versions 8.9 and 10.0). All treatments were performed with photon beams of 18 MV for CRT and of 6 MV for RA generated by either a Varian Clinac 2100 or by a TrueBeam linear accelerator equipped with a Millennium 120 MLC.

### Conformal radiation therapy

For all patients in the CRT cohort, the treatment planning CT scans were acquired without intravenous and oral contrast in free quiet breathing mode with a slice thickness of 3 mm. Patients were positioned supine, with the arms raised above the head. Immobilisation was granted by means of legs fixations. The treatment plans were designed and customized according to the characteristics of the individual case with multiple static fields (3–5 per plan), conformed to the target volumes with the multileaf collimator. Dose distributions were improved by using mechanical or virtual wedges. Image guidance was performed by means of paired orthogonal two-dimensional kilo-voltage images at the first fraction followed by similar procedures twice per week. Patient repositioning was performed whenever necessary.

The treatment of patients in the CRT group was performed with a sequential approach with a dose prescription of 45 Gy in 25 fractions to the pelvic PTV followed by a boost dose of 14.4 Gy in 8 fractions to ) PTV_boost (inclusive of eventual positive nodes). Plans were normalized to the isocenter as per ICRU62 [16] specifications.

### Volumetric modulated arc therapy

Starting with 2009, all anal SCC patients were treated with VMAT RA. In this subgroup of patients, CT scans with and without contrast intravenous and oral contrast were acquired in free quiet breathing mode with a slice thickness of 3 mm and used for treatment planning. Patients were positioned as for the CRT group. Image guidance for the RA group was performed by means of Cone Beam CT imaging (CBCT) before every treatment session. When necessary, treatment couch repositioning was performed after automatic matching of CBCT images to the reference planning CT, followed by manual refining. Matching was performed on bones and, when possible, on soft tissue structures (e.g. main blood vessels). The RA plans were optimized for each individual patients using 2–4 full coplanar arcs with a typical collimator rotation in the range of 10-30° or 80-85°.

With RA, the treatment was administered with a simultaneous integrated boost (SIB) approach. With SIB, both primary (PTV_boost) and pelvic (PTV) target volumes are treated simultaneously with different dose levels as easily achievable with the use of intensity modulated beams. The dose to the primary tumor and involved nodes was 59.4 Gy in 33 fractions (1.8 Gy per fraction), independently of the T stage. The dose to the elective nodal volume was 49.5 Gy in 33 fractions with 1.5 Gy per fraction. Plans were normalized to the mean CTV_boost dose as per ICRU83 [17] recommendations.

### Chemotherapy

Most of the patients received concurrent chemo-radiotherapy CH-RT. The indication and choice of chemotherapy regimen was left to the referring medical oncologist.

Concurrent CH was given to patients with tumors greater than 5 cm in size and/or with nodal involvement, when KPS status was >70. The most commonly used regimen was fluorouracil (FU) 1000 mg/m^2^/day for 4 day cycles (days 1–4 and 29–33) and mitomycin-C (MMC) 10 mg/m^2^ (maximum dose 20 mg) on days 1 and 29. Other regimens used during this time period included FU 1000 mg/m^2^/day for 4 day cycles (days 1–4 and 29–33) and cisplatin (cis-diamminedichloroplatinum(II) CDDP) 75 mg/m2 (days 1, 29) or FU 1000 mg/m^2^/day for 4 day cycles (days 1–4 and 29–33) given alone due to other co-morbidities.

Six HIV positive cases were treated with locally advanced anal SCC histologically confirmed and good performance status (PS 0–1) with concomitant chemo-radiotherapy and antiretroviral therapy. All 6 patients were immunologically stable in antiretroviral therapy.

### Evaluation

Dosimetric parameters of treatment plans were scored by means of dose volume histograms (DVH) analysis. This was done for all patients in the two cohorts.

Clinical evaluation during the course of treatment was performed weekly and included laboratory tests. Assessment of treatment response was performed, with reference to baseline conditions, at 3 and 6 months after treatment (with CT scan and proctoscopy/sigmoidoscopy) and then every 3–4 months. Late follow-up included a CT scan every 6 months while proctoscopy/sigmoidoscopy was performed at least once per year.

For the scope of the present analysis, acute and late toxicities were retrieved retrospectively from clinical charts as worst toxicity reported (per each domain). In general, toxicity assessment was performed at all follow-up visit. Toxicity was scored using the Radiation Therapy Oncology group (RTOG) and the Common Terminology Criteria for Adverse Events (CTCAE) v. 4.0 respectively. Evaluation of tumour response was defined according to the Response Evaluation Criteria in Solid Tumour (RECIST) v.1.1 [18].

Statistical analysis was performed by standard Kaplan-Meier and Fisher tests per each of the two cohorts (SPSS package, version 20.0). The Wilcoxon matched-paired signed-rank test was applied to evaluate the level of significance of the observed differences between the dose-volume metrics. The threshold for statistical significance was set at 0.05. The Mann–Whitney U test for independent samples was applied to assess the potential difference between the toxicity profiles.

## Results

### Patients and dosimetric characteristics:

Table 1 summarizes the demographic and clinical characteristics of the patients. Twenty-eight patients were treated with CRT and thirty-six with RA. Six patients (3 per group) had HIV positive status and all concluded the treatment receiving the entire prescription dose. One patient in the RA group had evidence of liver metastasis at diagnosis. CRT patients received the prescribed treatment in 26/28 cases. One patient interrupted the treatment after 31 fractions (acute skin toxicity of grade 3). All RA patients completed the prescribed treatment with the exception of one case interrupted after 32 fractions due to acute diarrhea toxicity (G2). Toxicity related treatment pauses longer than 3 days were registered and reported in Table 1 with a modest longer median duration in the CRT group.

**Table 1:** **Demographic and clinical characteristics**

	CRT		RA	
	n	%	n	%
**Total no of patients**	28	-	36	-
**Age, years**				
**Median**	59 [41–80]	-	65 [44–84]	-
**Sex**				
**Male**	9	32.1	7	19.4
**Female**	18	64.3	29	80.6
**HIV status**				
**Positive**	3	10.7	3	8.3
**Negative**	24	85.7	33	91.7
**HPV status**				
**Positive**	2	7.1	1	2.8
**Negative**	25	92.8	35	97.2
**T stage**				
**Tx**	0	0	2	5.6
**T1**	1	3.6	1	2.8
**T2**	18	64.3	21	58.3
**T3**	6	21.4	9	25.0
**T4**	3	10.7	3	8.3
**N stage**				
**Nx**	1	3.6	0	0
**N0**	12	42.9	23	63.9
**N1**	12	42.9	6	16.7
**N2**	1	3.6	2	5.6
**N3**	2	7.1	5	13.9
**M stage**				
**M0**	28	100	35	97.2
**M1**	0	0	1	2.8
**Stage**				
**X**	1	3.6	2	5.6
**I**	1	3.6	1	2.8
**II**	10	35.7	19	52.8
**III**	16	57.1	13	36.1
**IV**	0	0	1	2.8
**Chemotherapy**				
**FU/MMC**	11	39.3	29	80.5
**FU/CDDP**	16	57.1	2	5.5
**FU**	1	3.6	0	0
**None**	0	0	5	13.9
**Radiation therapy (33 fractions)**				
**Prescription (Gy)**	45/59.4		49.4/59.4	
**Duration median (days)**	49		51.0	
**RT break >3 days**	6	21	6	17
**Median break (days)**	10	-	7.5	-

**Figure 1:**
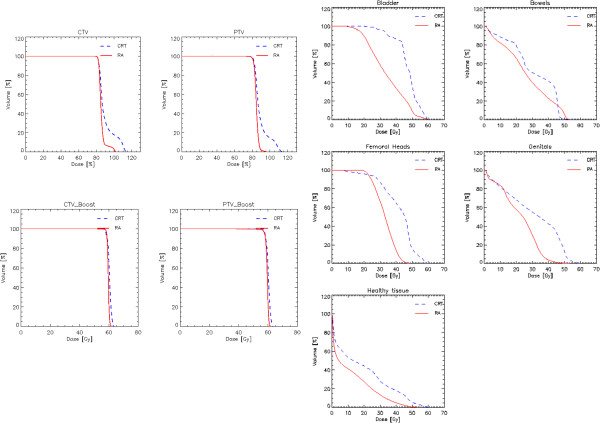
**Average DVH for the CTV, PTV and OARs for the two cohorts of patients.**

Table 2 reports the summary of the DVH analysis for all the patients for the various CTV, PTV and organs at risk. Figure 1 shows the average DVH for the corresponding structures comparing the two techniques (healthy tissue in the body volume in the CT scan minus the envelope of targets). The quantitative analysis of the data, revealed that both techniques achieved an high degree of target coverage in the absence of any statistically significant difference. Concerning organs at risk, statistically significant differences were observed for most of the structures. In more detail, the more modern technique based on rotational intensity modulation delivery (RA) allowed to respect on average the planning objectives. The conventional CRT approach resulted in a systematic failure in the fulfillment of the ideal dose-volume objectives.

**Table 2:** **Summary of the DVH analysis for the CTV, PTV and OARs for the entire cohort of patients**

Parameter	CRT		RA	
	**CTV**	**PTV**	**CTV**	**PTV**
**Mean (Gy)**	49.3 ± 1.3	48.8 ± 1.0	50.9 ± 0.7	50.2 ± 0.5
**D**_**1%**_ **(Gy)**	60.8 ± 1.2	60.4 ± 1.1	59.7 ± 0.6	54.4 ± 1.4
**D**_**99%**_ **(Gy)**	44.4 ± 0.6	43.4 ± 1.0	48.4 ± 0.6	46.9 ± 0.8
	**CTV_Boost**	**PTV_Boost**	**CTV_Boost**	**PTV_Boost**
**Mean (Gy)**	60.4 ± 1.1	60.2 ± 1.0	59.5 ± 0.3	59.3 ± 0.3
**D**_**1%**_ **(Gy)**	61.3 ± 1.3	61.5 ± 1.3	60.6 ± 0.4	61.0 ± 0.4
**D**_**99%**_ **(Gy)**	58.9 ± 0.8	57.9 ± 1.4	57.8 ± 1.6	55.5 ± 4.8
	**Bladder**			
**Mean (Gy)**	47.2 ± 5.6 (p = 0.02)		34.2 ± 6.6	
**V**_**40 Gy**_ **(%)**	86.4 ± 25.4 (p < 0.001)		33.8 ± 18.2	
	**Bowel**			
**Mean (Gy)**	30.8 ± 9.1		26.5 ± 6.8	
**V**_**40 Gy**_ **(%)**	41.4 ± 27.7 (p = 0.04)		22.9 ± 9.0	
	**Femoral heads**			
**D**_**1%**_ **(Gy)**	51.4 ± 7.2		43.8 ± 2.6	
	**Genitals**			
**V**_**40 Gy**_ **(%)**	42.6 ± 24.8 (p < 0.01)		22.1 ± 10.3	
	**Healthy tissue**			
**Mean (Gy)**	18.9 ± 4.4 (p = 0.01)		11.9 ± 1.6	
**V**_**10 Gy**_ **(%)**	53.6 ± 11.9 (p = 0.03)		41.4 ± 6.1	

Data are reported as average values plus or minus standard deviation.

Dx%: dose received by at least x% of the volume; Vx%: volume receiving at least x% of the dose. p values from independent samples test have been reported only for cases with p < 0.05.

For the bladder, the constraint of V_40 Gy_ < 50% was improved of about 15% with RA while it was severely violated for the CRT patients. V_40 Gy_ < 30% for the bowel was improved of about 8% with RA; V_40%_ < 25% for the genitals was slightly improved with RA while it was almost doubled with in the CRT cohort; D_1%_ < 50 Gy for the femoral heads safely achieved for RA and almost for CRT.

### Survival and local control

The median follow-up of the patients was: 68.5 (range: 6–93) and 19.0 (range: 7–59) months for the CRT and RA groups respectively.

**Figure 2:**
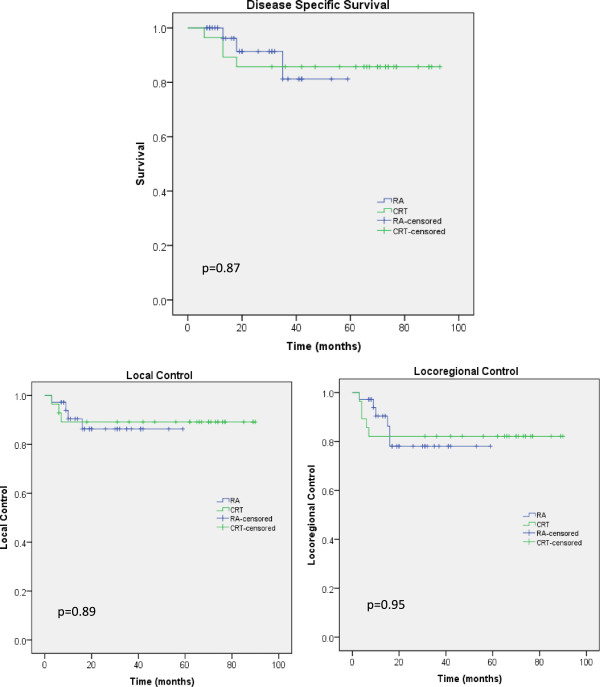
**Disease specific survival, local control and loco-regional control curves for the two cohorts of patients.**

Figure 2 shows the Kaplan-Meier graphs for the Disease Specific Survival, DSS, (panel a), for the Local Control (panel b) and for the Loco-regional Control (panel c) for the two groups of patients. No statistical significance was observed in the difference between the groups. Median survival was not reached while the mean actuarial survival resulted of 52.8 ± 3.3 months (95% C.L.: 46.3-59.3) for RA and 81.5 ± 5.3 months (95% C.L.: 71.0-91.9) for CRT. DSS at 2 years was: 85.7% and 91.3% for CRT and RA and DSS at 5 years was: 85.7% and 81.2% (at 59 months for RA) respectively.

Five years Loco-regional control was of 78.1% and 82.1% for RA and CRT respectively. Complete response was achieved in 54 patients (24 and 30 in the CRT and RA groups respectively); 90.7% of these received CH-RT. All HPV patients achieved complete response. All but one HIV positive patients obtained complete response. This case was treated with CRT and presented both local and regional relapse. Local failure was observed in 3 patients per group (10.7% for CRT and 8.3% for RA); regional failure in 4 patients per group (14.3% and 11.1% respectively); distant failure was observed in 3 patients for CRT (10.7%) and in 2 patients for RA (5.6%). Abdomino-perineal resection (APR) was necessary in 3 patients presenting with local failure after CH-RT.

Three of the CRT patients had local relapse associated with regional relapse. In the group of patients treated with RA, one achieved a partial response and required an abdominal resection 3 months after completion of radio-therapy. Three further patients had local relapse, associated with regional relapse in two cases. In other 2 patients there was regional failure without local relapse. One patient experienced abdominal lymph-adenopathy and liver metastases without local and regional relapse.

### Toxicity

Table 3 reports data for acute toxicity in the gastro-intestinal (GI) and genitor-urinary (GU) tracts as well as for the skin reactions. RA treatments lead to a reduction of the absolute incidence of higher grade events. In fact, grade 2–3 toxicity reduced of ~20% for GI (p = 0.06), of ~6% for GU (but not statistically significant) and of ~7% for the skin (with p = 0.05). Grade 1 toxicity reduced of ~10% for GI. No grade 4 toxicity was observed for any endpoint. Nevertheless, the distributions of toxicity across the therapy categories, resulted the same on the basis of the results of the two independent samples Mann–Whitney U test for the tree domains. Specifically, p resulted 0.06, 0.3, 0.06 for the GI, GU and skin toxicity distributions respectively.

**Table 3:** **Observed acute toxicity**

	CRT		RA	
	n	%	n	%
**Gastro-intenstinal**				
**G0**	0	0	0	0
**G1**	3	10.7	11	30.6
**G2**	20	71.4	22	61.1
**G3**	5	17.9	3	8.3
**G4**	0	0	0	0
**Genito-urinary**				
**G0**	8	28.6	16	44.4
**G1**	9	32.1	8	22.2
**G2**	11	39.3	12	33.3
**G3**	0	0	0	0
**G4**	0	0	0	0
**Dermatologic**				
**G0**	0	0	0	0
**G1**	5	17.9	9	25
**G2**	12	42.8	22	61.1
**G3**	11	39.3	5	13.9
**G4**	0	0	0	0

Most of patients completed RT without interruptions. Six patients had treatment break >3 days, 21% and 17%, in both groups, 3D and VMAT respectively. Twenty two CRT patients completed therapy without breaks or <3 days versus thirty cases treated with VMAT.

The reasons for all treatment break in our cases were: gastrointestinal toxicity (8 cases in VMAT group and 6 in CRT group), dermatological toxicity (4 and 6 patients in VMAT and CRT groups), genitourinary toxicity (1 and 2 patients in VMAT and CRT groups) or haematological toxicity (5 cases in both groups). No significant differences were found in treatment breaks.

Late toxicity was scored as follows: 5/36 (14%) and 3/36 (8%) patients had GI toxicity of grade 1 and grade 2 respectively in the RA group. This was observed in 1/28 (4%) and 4/28 (14%) in the CRT group. GU late toxicity was observed only in 4/28 (14%) patients of the CRT group: 3/28 (11%) had G2 and 1/28 (4%) had G1.

## Discussion

The effectiveness of chemo-radiation therapy for anal cancer has been demonstrated by several randomized trials [19–21]. The most recent trials [22, 23] suggested that CH-RT with FU and MMC should remain the standard of care. However, concurrent chemo-radiation is associated with relevant acute gastrointestinal, genitourinary, dermatological toxicities when conventional radiation therapy techniques are used.

Both the Long-Term Update of RTOG 98–11 Phase III [24] and the ACT II trial [23] showed that toxic effects during chemo-radiation were similar in the mitomycin and cisplatin treatment groups. Grade 3–4 hematologic toxicity was more common in the mitomycin group. The rate of acute non-hematologic grade 3 or 4 toxicity was 74% in both groups according to the RTOG trial. As a consequence, prolonged treatment breaks were necessary and have been shown to negatively affect local control [25, 26]. The data presented in this study showed that treatment interruptions had no significant impact on local control while in the study of Graf [25] it was shown that patients with overall treatment time >41 days had 5 yr LC of 58% versus 79% if overall treatment time was <41 days (p = 0.04).

Recently, there has been increasing attention on the use of IMRT. Bazan [4] and Choung [6] directly compared IMRT to CRT. In both series the use of IMRT was associated with decreased toxicity and a consequent reduction in the treatment breaks. Dasgupta [3] focused on the outcomes of IMRT versus CRT and the results demonstrated that in particular LRC was not compromised by more conformal radiotherapy.

In the present study, 64 consecutive patients were evaluated after treatment with radiotherapy alone or concurrent chemotherapy with CRT or VMAT. Tumor characteristics were similar in both groups. In the VMAT group, one patient had stage IV at diagnosis with single liver metastases. This patient was treated neoadjuvantly with a combination of cisplatin and fluorouracil [27, 28] chemotherapy. After 6 cycles of systemic therapy at completed response, the patient was enrolled for definitive radio-chemotherapy with fluorouracil and mytomicin regimen [19, 22].

Our study had a median follow-up of 68.5 months (range 6–93) and 19.0 months (range 7–59) for CRT and VMAT groups respectively, in line with the previously reported studies. Although the median follow-up for the VMAT cohort was shorter, the rates of higher grade toxicities were lower than among the CRT cohort.

There might be some potential factors to consider in addition: the different prescription doses in the two groups and the different chemotherapy regimens followed. These might obviously contribute to the different toxicity profiles observed in the data. In addition, in a retrospective analysis, toxicity reports tend to be inferior than what achieved from prospective investigations.

Three HIV positive patients were treated with CRT and 3 with VMAT while two HPV positive cases underwent CRT and one patient VMAT RA. Both HIV and HPV positive cases had comparable acute and late toxicity versus non HIV/HPV patients. Albeit this study is limited by low patient numbers, its results support previous findings that high-dose EBRT in HIV-positive patients has durable biochemical control with limited toxicity in line with that of non–HIV-positive patients [29].

Analyzing the PTV coverage, the data resulted comparable between the two techniques, but the normal healthy tissue sparing was more pronounced in the VMAT plans, in particular for the bladder, the external genitalia and the femoral heads.

Survival data are similar to earlier studies [3, 4, 7, 10]. Our cohort treated with VMAT had a DSS at 2 yrs, LCC and LRC rates of 85.7%, 86.3% and 78.1% respectively comparing with CRT cohort with 91.3% of 2 yrs DSS, 89.1% of LCC and 82.1% of LRC.

Although VMAT does not appear to improve the survival outcomes comparing to the CRT, its advantage is principally the reduction of the severe RT-related toxicity. The future prospective should consider dose–escalation strategies to improve the disease-related outcomes.

## Conclusion

The treatment of anal carcinoma patients with VMAT (RapidArc) was evaluated in a retrospective comparison against conformal therapy and resulted in the same disease specific survival and loco or loco-regional control. Improved profiles of toxicity were observed for the patients treated with RapidArc.
